# Effects of intravenous lidocaine on hypoxemia induced by propofol-based sedation for gastrointestinal endoscopy procedures: study protocol for a prospective, randomized, controlled trial

**DOI:** 10.1186/s13063-022-06719-6

**Published:** 2022-09-24

**Authors:** Xiu-Ru Qi, Jing-Yi Sun, Li-Xin An, Ke Zhang, Fu-Shan Xue

**Affiliations:** Department of Anesthesiology, Beijing Friendship Hospital, Capital Medical University, Beijing, China

**Keywords:** Lidocaine, Hypoxemia, Painless gastrointestinal endoscopy, Propofol, Sedation

## Abstract

**Background:**

Oxygen-desaturation episodes, blood pressure drops, and involuntary body movement are common problems that occur in propofol-based sedation in the procedure of painless gastrointestinal (GI) endoscopy. As a widely used analgesic adjuvant, intravenous lidocaine can reduce the consumption of propofol during ERCP or colonoscopy. However, it is still unknown how lidocaine affects the incidence of oxygen-desaturation episodes and cardiovascular events, and involuntary movement during painless GI endoscopy. Therefore, we aimed to assess the effectiveness and safety of intravenous lidocaine in propofol-based sedation for GI endoscopy.

**Methods:**

We will conduct a single-center, prospective, randomized, double-blind, saline-controlled trial. A total number of 300 patients undergoing painless GI procedures will be enrolled and randomly divided into the lidocaine group (Group L) and the control group (Group C). After midazolam and sufentanil intravenous injection, a bolus of 1.5 mg/kg lidocaine was immediately injected and followed by a continuous infusion of 4 mg/kg/h in the lidocaine group, whereas the same volumes of saline solution in the control group. Then, propofol was titrated to produce unconsciousness during the procedure. The primary outcome will be the incidence of oxygen-desaturation episodes. Secondary outcomes will be the incidence of involuntary body movement, discomfort symptoms, propofol consumption, endoscopist, and patient satisfaction.

**Discussion:**

Propofol-based deep sedation without intubation is widely used in painless GI endoscopy. However, adverse events such as hypoxemia often occur clinically. We expect to assess the effect of lidocaine on reducing the incidence of oxygen-desaturation episodes, cardiovascular events, and involuntary body movement. We believe that the results of this trial will provide an effective and safe method for painless GI endoscopy.

**Trial registration:**

Chinese Clinical Trial Registry ChiCTR2100053818. Registered on 30 November 2021.

**Supplementary Information:**

The online version contains supplementary material available at 10.1186/s13063-022-06719-6.

## Background

Gastrointestinal (GI) endoscopy has been considered as the most accurate diagnosis for screening and diagnosing gastrointestinal cancer early. With the increasing demand of patients for comfortable medical care, procedure sedation and anesthesia (PSA) has become a widespread method to relieve patients’ anxiety, discomfort, and pain during GI endoscopy. Although, the proportion of PSA for GI endoscopy varies in different countries because of different medical conditions and habits [1–3]. Drugs used for PSA, management of their side effects, monitoring, and recovery have been paid more and more attention by endoscopists and anesthesiologists. Especially in the combined examination of gastroscopy and colonoscopy, due to the long operation time and procedure stimulation, special events such as respiratory depression, limb movement, and arrhythmia occur from time to time. For gastrointestinal endoscopy, anesthesia and other issues are particularly important.

Propofol is the main sedative hypnotic drug recommended for GI endoscopy sedation because of its rapid action and short half-life pharmacologic characteristics. However, hypoxemia is the most common adverse event that occurs during propofol sedation [4–6]. If hypoxia is severe or lasts for a long time, patients may have the risk of arrhythmia, permanent neurologic damage, cardiorespiratory arrest, or death [7–9]. Therefore, efforts about reducing the incidence of hypoxia and severe hypoxia are necessary during painless endoscopy procedures. The independent risk factors for hypoxemia include [3] aging, high body mass index, sleep apnea syndrome, operation time, and propofol consumption, and propofol is associated with the high risk of cardiopulmonary depression events in a dose-dependent manner [10]. Therefore, the application of adjuvant drugs with less side effects on respiratory and cardiac inhibition, so as to reduce the dose of propofol, is beneficial for painless GI endoscopy. For example, benzodiazepines and opioids used with propofol could significantly decrease propofol requirements [11], but hypotension and hypoxemia still occur [12, 13]. Ketamine used with propofol reduced propofol consumption and cardiopulmonary depression events, but ketamine also produced schizophrenia and dissociative states [14]. Although dexmedetomidine possesses anxiolytic and sedative properties without respiratory side effects during the endoscopy, it causes prolonged hypotension and bradycardia [15–17].

In clinical practice, intravenous lidocaine is usually used to treat arrhythmia, but it has also been proven to reduce visceral pain, decrease opioid consumption, accelerate the recovery of postoperative intestinal function and promote rehabilitation after visceral surgery. It has been widely used as an adjuvant analgesia drug at present [18, 19]. Owing to its some properties, such as antinociceptive action and the increased ventilatory response to CO_2_ [20], intraoperative application of lidocaine can reduce the dosage of propofol, reduce the number of involuntary body movements during surgical stimulation, and reduce the occurrence of other adverse respiratory events such as hypoxemia [21–23]. Liu. et al found that intravenous lidocaine can significantly decrease propofol requirements during ERCP [23]. However, the effect of intravenous lidocaine on the incidence of oxygen-desaturation and cardiovascular events, and involuntary movement in patients who perform gastroscopy and colonoscopy joint examination is still unknown.

This prospective, single-center, double-blind, randomized, placebo-controlled trial is going to verify the effects of intravenous lidocaine, as an adjuvant to propofol-based painless GI endoscopy, on oxygen desaturation and involuntary body movements.

## Methods/design

### Objectives and design

**Fig. 1:**
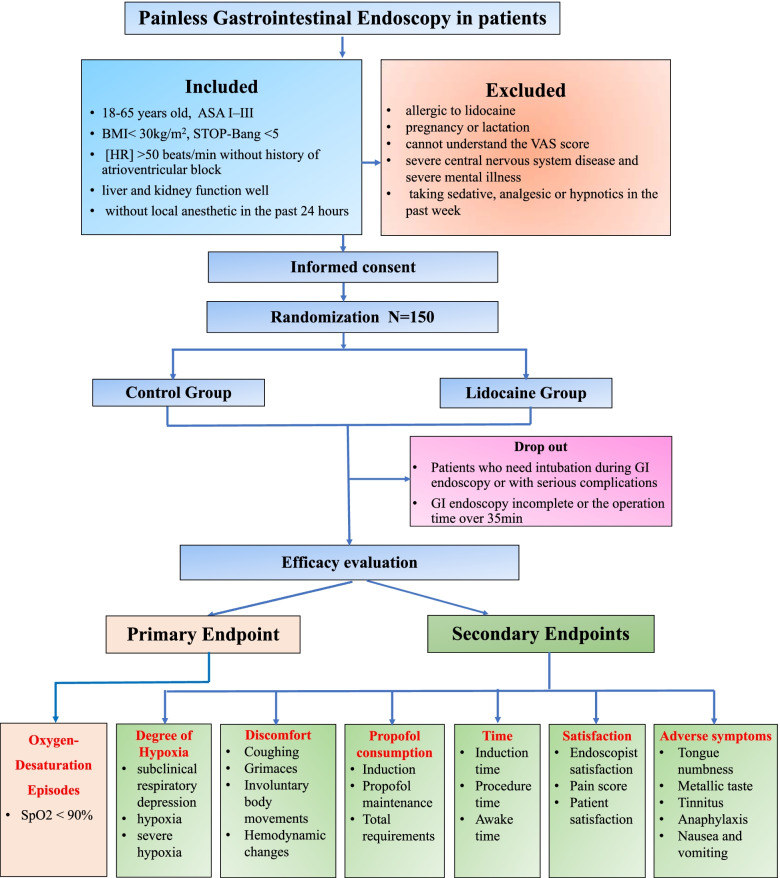
Consort diagram

This study is a single-center, double-blind, randomized, placebo-controlled trial, that aims to test the hypothesis that lidocaine is an ideal adjuvant for painless GI endoscopy based on propofol with less episodes of oxygen desaturation and involuntary body movements. 300 patients will be randomly divided into either the lidocaine group or the control group at a ratio of 1:1 (see Consolidated Standards of Reporting Trials [CONSORT] diagram, Fig. 1).

This trial will be conducted at the Beijing Friendship Hospital affiliated to Capital Medical University, China, according to the WMA of the Declaration of Helsinki and the CIOMS Principles of the International Guidelines for Biomedical Research Involving Human Subjects. The protocol has been approved by the Ethics Committee of the Beijing Friendship Hospital Affiliated to Capital Medical University (the approval number is 2020-P2-159-02) and has been registered at the Chinese Clinical Trial Registry (Chictr) (the registration number is ChiCTR2100053818). The detailed information on the trial registry is in Additional file 2. Compensation and post-trial care will be given to those who suffer harm from trial participation according to hospital regulations.

### Blinding and randomization

This is a double-blind study. Only the nurse (A) who is responsible for preparing the trial drug knows the grouping information automatically generated by a computer. She (A) will generate the allocation sequence. The chief anesthesiologist (B) will enroll patients and assign participants to interventions. Patients, endoscopists, anesthesiologists, and data collection observers will be all blinded to the group allocation. When visiting patients in the anesthesia preparation room, the chief anesthesiologist (B) will screen the patients according to inclusion/exclusion criteria. The anesthesiologist will explain the process, benefits and risks of this trial, and the collection of participant data to the patients who meet the inclusion criteria. Informed consent will be signed after consent is obtained by the anesthesiologist (B). Then, the anesthesiologist will inform the nurses (A) who are responsible for random grouping of the patient’s inclusion information, obtain the configured and covered test drug from the nurses, and begin to implement anesthesia.

### Study population

Patients scheduled for painless gastrointestinal endoscopy will be screened and recruited during routine preoperative evaluation. Participants will be eligible if they meet the following inclusion criteria: 18–65 years old, at the American Society of Anesthesiologists (ASA) Physical Statuses I–III, BMI< 30kg/m^2^, STOP-Bang <5, heart rate [HR]>50 beats/min without history of atrioventricular block, liver and kidney function well, without local anesthetic in the past 24 hours, without analgesics and hypnotics in the past 7 days, volunteer to participate in this research and sign the informed consent. In addition to full communication, some small gifts like incentives will be provided to patients to facilitate the enrollment.

Exclusion criteria are as follows: patients aged >65 years or <18 years, have participated in other clinical trials within the past 4 weeks, allergic to lidocaine, pregnancy or lactation, cannot understand the VAS score and cannot be scored, severe central nervous system disease and severe mental illness, taking a sedative, and analgesic or hypnotics in the past week are considered unsuitable to participate in this study by the investigator.

### Standard procedure

**Fig. 2:**
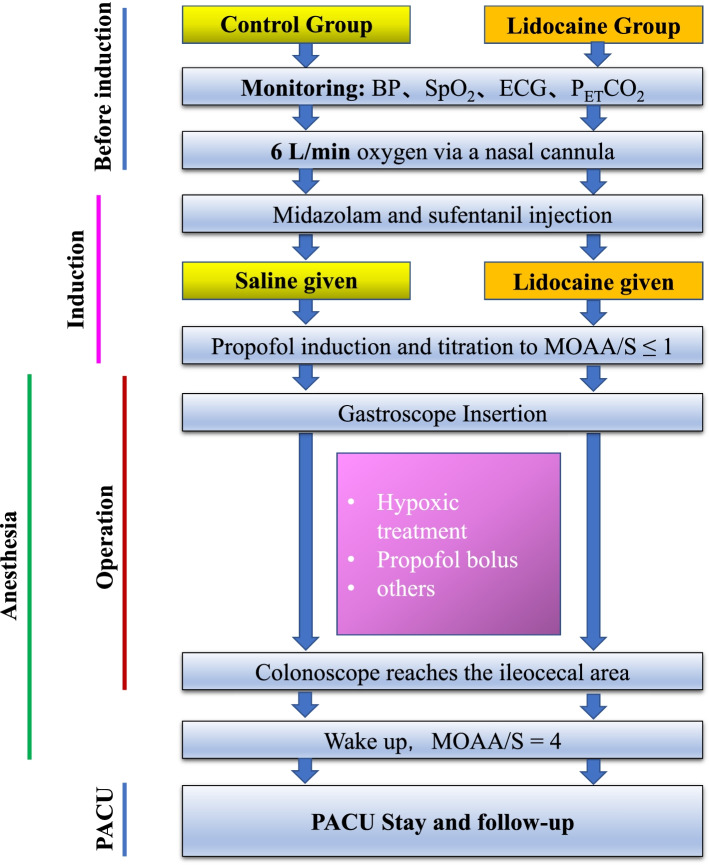
Perioperative prtocol

To avoid interfering with the trial intervention, the deep sedation based on propofol is conducted by a fairly fixed team of anesthesiologists in accordance with the clinical routine. The following strategies are recommended (Fig. 2):

1. All patients scheduled for painless gastroscopy and colonoscopy will be evaluated and screened at the Anesthesiology Evaluation Clinic to assess their risk for sedation according to the ASA guidelines.
2. Participants will be filtered in accordance with the inclusion and exclusion criteria before the procedure. A nurse is responsible for preparing the medicine according to an envelope with the smallest sequential number after the intravenous catheterization.
3. According to clinical routine, upon admission to the endoscopic room, following parameters will be continuously monitored: electrocardiogram, noninvasive blood pressure, peripheral oxygen saturation, respiratory rate, and waveform capnography.
4. With patients in a left lateral position, 6 L/min oxygen via a nasal cannula was supplied.
5. At the moment of 60 s before the administration of propofol, noninvasive blood pressure will be measured and recorded, midazolam 0.02 mg/kg, sufentanil 0.1 μg/kg, and then the intervention medicines will be given intravenously in turn to patients of both groups.
6. Induction: An initial bolus of propofol (adjusted to the patient’s age: 1.0 mg/kg for age >50; 1.5 mg/kg for age <50) is administered. Then Modified Observer’s Assessment of Alertness/sedation (MOAA/S) will be evaluated [24]. A repeated dose of 10–20 mg propofol is injected if MOAA/S >2. Until the patients’ MOAA/S score is ≤1, the gastroscope will be inserted by a skilled endoscopist, who have completed more than 500 procedures, less than 1000.
7. Sedation maintains: During the procedure, additional propofol (0.5 mg/kg) is repeated in case any discomfort occurs, including coughing, grimaces, involuntary body movements, hemodynamic changes (increase in SBP ≥20 mmHg, HR ≥20 beat/min), or MOAA/S scale >1.
8. When the colonoscope reaches the ileocecal area, the intravenous injection of intervention drug will be stopped. When the operation is completed, all patients will be transferred to the post-anesthesia care unit (PACU).

All dates related to the procedures applied will be collected by an observer and analyzed by a statistics specialist, both of whom do not clear the assignments. All sedation and related treatment should comply with clinical routines. Besides, staff should remember the possibility of intravenous lidocaine toxicity, lipid emulsion 20% should be available wherever it is used.

#### Intended sedation target

Once the induction starts, the anesthesiologist will keep an eye on the patient in order to continually evaluate the depth of sedation and keep the MOAA/S score ≤1:

- Level 5: Responds readily to name spoken in a normal tone,
- Level 4: Lethargic response to name spoken in a normal tone,
- Level 3: Responds only after the name is called loudly and/or repeatedly,
- Level 2: Responds only after mild prodding or shaking,
- Level 1: Responds only after painful trapezius squeeze,
- Level 0: No response after painful trapezius squeeze.

### Intervention

The 300 eligible patients will be randomly allocated into two groups at a 1:1 ratio: lidocaine group (group L, *n* = 150), and normal saline group (group C, *n* = 150) according to the random number sequence. After midazolam and sufentanil given, the intervention medicines will be administrated to patients immediately by the blinded anesthesiologist in charge, and the initial dose is 0.15 ml/kg, the following infusion speed is 0.4 ml/kg/h. For each group, participants will receive intervention as follows:

- ♦ Group L: Lidocaine will be administrated at 1.5 mg/kg (0.15ml/kg, 10mg/ml) as initial dose, then followed by 4mg/kg/h (0.4 ml/kg/h) infusion until the ileocecal area is exposed.
- ♦ Group C: Normal saline will be administrated at 0.15 ml/kg, and the following infusion speed is 0.4 ml/kg/h, the same volume with lidocaine, until the ileocecal area is exposed.

There will be no special criteria for modifying the interventions. In case the severe adverse events appear during the operation, the inventions will be terminated immediately, and the rescue procedure will be started.

#### Hypoxic treatment

**Fig. 3:**
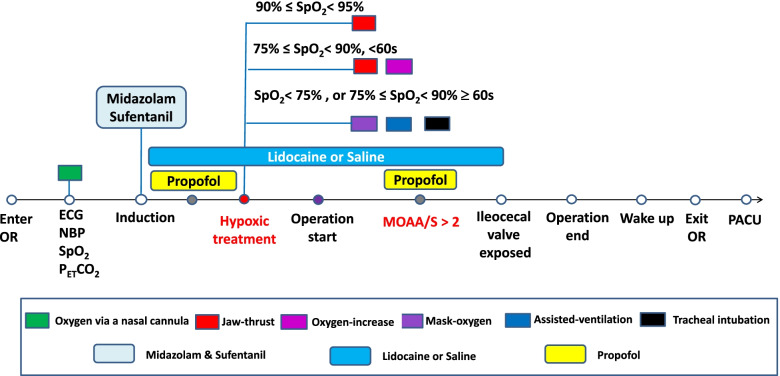
Administration and hypoxic treatment protocol

During the procedure, when the trends in hypoxemia (90% ≤ SpO_2_< 95%) appear, it will be improved with using the jaw-thrust maneuver. If hypoxia (75% ≤ SpO_2_< 90% for <60 s) develops, it will be treated with both using the jaw-thrust maneuver and increasing the oxygen from 6 to 10 L/min. Once severe hypoxia (SpO_2_< 75% or 75% ≤ SpO_2_< 90% for ≥60 s) occurs, patients will be rescued by assisted mask ventilation, even tracheal intubation if necessary [25] (Fig. 3).

### Study endpoints

The primary endpoint of the study is the incidence of oxygen-desaturation episodes (ODE). Oxygen-desaturation episodes are defined that the time of SpO_2_< 90% for more than 10 s [25]. Once the patient has hypoxia, we will start to time and record its duration. Once the time of SpO_2_< 90% is longer than 10s, we consider this case as having ODE. We will record and count the number and duration of hypoxia of different degrees in a case.

The secondary endpoints are as follows: ① The incidence of subclinical respiratory depression (the time of SpO_2_< 95% for more than 15s), hypoxia (the time of 90% ≤ SpO_2_< 95% for more than 10 s), and severe hypoxia. ② The incidence of patients with discomfort (coughing, grimaces, involuntary body movements, and hemodynamic changes) after propofol induction to awake. ③ The propofol dose of induction, the propofol dose of maintenance, and the total propofol dose requirements. ④ The induction time, the time from induction to first propofol bolus, procedure time, awake time. ⑤ The endoscopist satisfaction, pain score, and patient satisfaction. ⑥ The adverse symptoms and the related treatments during the procedure will be also recorded. ⑦ Lidocaine-related side effects also need to be recorded and related treated, such as tongue numbness, metallic taste, tinnitus, anaphylaxis, nausea, and vomiting.

### Definitions

The subclinical respiratory depression is defined as 90% ≤ SpO_2_< 95%, hypoxia is 75% ≤ SpO_2_< 90% for more than 10 s, and the severe hypoxia is SpO_2_< 75% or 75% ≤ SpO_2_< 90% for 60 s. The required airway management is also recorded.

The discomfort symptoms include coughing, grimaces, involuntary body movements, and hemodynamic changes (increase in SBP 20 mmHg, HR 20 beats/min).

The propofol dose of induction is explained as the propofol consumption making the MOAA/S score ≤1 firstly.

Induction time referred to the time from injection of the first sedative to the MOAA/S score ≤1.

Procedure time is the time between insertion of the gastroscope and colonoscope peeps into the ileocecal area.

The awake time is the time between peeping the ileocecal area and the MOAA/S score ≥3.

Endoscopist satisfaction, patient satisfaction, and patient pain will be measured by a 10-point VAS that was 0 to 10 cm wide, with 0 representing extremely unsatisfied and no pain, while 10 representing excellent satisfaction and sharp pain.

### Date collection and management

Before surgery, patients’ demographic, clinical, and procedural characteristics will be collected carefully by including (1) demographic variables such as sex, age, height, weight, diagnosis, comorbidities, history of anesthesia and surgery, history of cigarette smoking, history of alcohol intake; (2) ASA physical status; (3) factors of difficult mask ventilation: male, age>55 years, snoring, no teeth, beard, anterior and posterior cervical mobility, mandibular mobility; (4) STOP-Bang score; and (5) SpO_2_ without inhaling oxygen.

During the sedation period, the following data will be collected: (1) heart rate, SpO_2_, blood pressure, and arrhythmia; (2) requirement of propofol; (3) the duration of induction, operation, and awake up; (4) details and related treatment of hypoxia.

After the patient wakes up, endoscopist satisfaction, patient satisfaction, and patient pain will be measured, and adverse symptoms recorded and kept on case report forms (CRF). The CRF will be available on the clinical trial registration website of this trial (http://www.chictr.org.cn/showproj.aspx?proj=56859).

The scientific research management committee (SRMC), is consisting of a blinded anesthesiologist, a scientific researcher, and a statistician. The function of the SRMC is data analysis and management, outcome adjudication, and protocol improvement. All data collection will be completed and locked by the blinded anesthesiologist and the follow-up doctor. Any non-protocol adherent cases will be retained, such as operation failed, those that cannot complete the primary endpoint assessment, and those that do not meet the standard procedures. When each group completes almost half of the cases, unblinding and interim analysis will be conducted by the statistician of SRMC. If the interim analysis after unblinding is consistent with the hypothesis, the test will be continued. If it is inconsistent or even contrary, the expert committee will be consulted to decide whether to continue the trial, terminate the trial, or expand the sample size. At the end of the trial, the original data and results will be submitted to SRMC, and the electronic information will be entered in a password-protected mailbox, they will not be shared to the public until the results are published. All original records (medical record information, informed consent, CRF, and related files) will be stored and saved for 10 years to promote participant retention and complete follow-up, and then destroyed in accordance with the hospital standards.

### Study dropoutsges

All subjects could withdraw from the trial at any time and for any reason. Moreover, any patient could be terminated by the anesthesiologist or endoscopist at any time, believing it is in the best interest of the patient. The reasons and circumstances for study discontinuation will be recorded in the CRF. First, all data will be analyzed according to the intention to treat (ITT) principle. Secondly, the data will be analyzed according to the protocol.

### Sample size calculations

The primary endpoint of the study is the incidence of patients with oxygen-desaturation episodes. From our pilot study, the incidence of oxygen-desaturation episodes in the control group was about 50%. We hypothesize that compared with the control group, the lidocaine group can reduce the incidence of deoxygenation saturation by 30%. We estimate the minimal sample size of 134 patients by using the Z-test (Unpooled) with PASS 15.0 software, with a 90% power at a 2-sided significance level of *P*=0.05. So, we will enroll 150 patients in each group finally considering 10% of possible dropouts in our study.

### Statistical analysis

The statistical analysis will be performed using the SPSS 23.0 software by the statistician. Kolmogorov-Smirnov test will be used to assess data normality. Categorical variables are expressed as numbers and percentages, and continuous variables are expressed as mean (standard deviation, SD) or median and interquartile range (IQR). Student t-test or Mann-Whitney *U*-test will be used to determine the significance of continuous parametric data, such as the propofol dose of induction, the propofol dose of maintenance, the total propofol dose requirements, the induction time, the time from induction to the first propofol bolus, procedure time, awake time, the endoscopist satisfaction, pain score, and patient satisfaction. Wilcoxon rank sum test will be applied to nonparametric data. For categorical variables, such as the primary endpoint. incidence of oxygen-desaturation episodes, subclinical respiratory depression, hypoxemia, and severe hypoxia between the two groups, we will apply the *χ*^2^ test or the Fisher’s exact test. A repeated-measures analysis of variance was used to analyze repeatedly measured data such as SBP and HR. Calculate the number of 95% confidence interval (CI) treatments needed to evaluate the effect of lidocaine on the primary endpoint, the incidence of oxygen-desaturation episodes.

Univariate logistic regression analysis was initially used to identify possible risk factors for the occurrence of deoxygenation saturation episodes. The variance expansion factor >2.5 (or high correlation coefficient >0.7) will be used to determine that there is multicollinearity between the relevant parameters with a *P* value ≤ 0.1 in univariate analysis. If there are multicollinearity variables, the variables will be selected according to the clinical significance. Then, multivariable logistic regression will be performed to identify risk factors associated with deoxygenation saturation episodes using remaining covariates with a *P*-value ≤ 0.1 for univariate analysis. The backward elimination method is used for stepwise selection without additional steps to change covariates to develop further risk prediction models. The Hosmer-Lemeshow goodness-of-fit test is used to compare the possibility of estimated results with observed results. All *P* values are two-sided; *P* < 0.05 will be considered statistically significant. In the regression analysis, if it is found that there are significant differences between the two groups in the factors that have a significant impact on the primary outcome, we will carry out a subgroup analysis separately. The two groups carried out regression analysis on the primary outcome separately, trying to find out the possible influencing factors.

The follow-up time of this study is at the end of PACU, so there will be no follow-up failure and the missing data will be little. In order to avoid the loss of data records caused by time constraints, for example, the duration of SpO_2_ < 90%, we will use a mobile phone to record the whole process during the procedure to supplement the data that has not been recorded. If there is still missing data, the mean imputation method will be used. In case of study dropout, these cases will be reported. ITT includes all population who is treated, including those with missing data obtained by the mean imputation method. Per-protocol refers to the population with complete data records and no missing data. Both ITT and per-protocol analysis will be performed. For ITT analysis, data from all patients in the randomization group will be processed. If a significant proportion of patients did not receive a random intervention or lost follow-up, a per-protocol analysis will be performed to evaluate the primary outcome.

## Discussion

This is a prospective, double-blinded, randomized, placebo-controlled, and single-center trial. It aims to confirm whether intravenous lidocaine contributes to reducing the incidence of oxygen-desaturation episodes and discomforts during propofol-based painless gastrointestinal endoscopy.

Deoxygenation events are well-established risks and complications during deep sedation procedures, with high incidences between 12% and 33% [6, 12, 25–29]. Differences could be explained by aging, high body mass index, sleep apnea syndrome, operation time, definition of deoxygenation, oxygen inhalation conditions, and the depth of sedation. We believe the present protocol is appropriate for several good reasons and could well avoid the influence of other factors except for lidocaine on the occurrence of deoxygenation. First, the definition is clear: according to most studies about deoxygenation, we define that SpO_2_< 90% for more than 60s is Oxygen-desaturation episodes. In addition, we also defined and recorded the different degrees of hypoxia. The subclinical respiratory depression is defined as 90% ≤ SpO_2_< 95% [6], hypoxia is defined as 75% ≤ SpO_2_ < 90% for <60 s, and severe hypoxia is SpO_2_< 75% for any duration or 75% ≤ SpO_2_< 90% for ≥60 s in accordance with the suggestion of the World SIVA International Sedation Task Force [30]. Otherwise, subclinical respiratory depression time will be recorded and analyzed to substitute with the difficulty of measuring the partial pressure of exhaled carbon dioxide (P_ET_CO_2_) and the subjectivity of observing the chest rising. Second, the intended sedation target is evaluated and aligned with the MOAA/S scale [24], meeting the ASA continuum of sedation. Besides, demographic characteristics, level of endoscopists, endoscopic equipment, injection time and speed of drugs, and oxygen inhalation conditions are all consistent.

Various adjunctive agents, diphenhydramine, promethazine, or droperidol, have been recommended in combination with conventional sedative drugs in select clinical circumstances [3]. Interestingly, intravenous lidocaine may be another adjunct to propofol-mediated sedation, with the beneficial properties of propofol-sparing effect [21–23], suppressing secondary hyperalgesia [31, 32], and increasing ventilatory response to CO_2_ [20]. However, it is still lacking the study on the incidence of oxygen-desaturation episodes during lidocaine adjuvant to propofol sedation, especially in the GI endoscopy. Simple gastroscopy or colonoscopy is not feasible for continuous administration of lidocaine because the operation time is short. However, when gastroscopy and colonoscopy are combined, intravenous lidocaine adjuvant administration becomes possible. In fact, with the gradual improvement of people's understanding of gastrointestinal endoscopy and the demand for comfortable medical treatment, but also for the convenience of patients, the joint examination of gastroscopy and colonoscopy has become more and more common in China. This makes it convenient and reasonable for intravenous infusion of lidocaine. In the present protocol, patients are given a bolus of lidocaine 1.5 mg/kg followed by a continuous infusion of 4 mg/kg/h. This dose of lidocaine injection for 30 min did not cause the toxic plasma [32].

It is a common complication of propofol-based sedation that body movement reaction occurs during colonoscopy. Body movement will not only reduce the satisfaction of endoscopists, but also lead to the risk of bleeding and even perforation. In our preliminary experiment, it was found that the incidence of body movement decreased significantly in patients with intravenous infusion of lidocaine. In the study by Jing Liu [23], the incidence of involuntary movement was 41.7% in the control group, while significantly reduced to 12.5% in the lidocaine group, consistent with our pilot study. In addition, propofol dosage, operation time, awake time, incidence of discomforts and its details, endoscopists’ satisfaction, patients’ satisfaction will be all recorded and analyzed.

In conclusion, we believe that this study will verify the hypothesis that intravenous lidocaine is contributed to reduce the incidence of deoxygenation events and involuntary body movement during propofol-sedation in GI endoscopy. Lidocaine may be an ideal adjuvant to propofol-mediated sedation.

### Trial status

The first participant was enrolled on December 1, 2021, and the first version was developed on September 1, 2020. The recruitment will be completed on December 31, 2022. Shown above is the second version whose protocol was revised for the following reasons: redefinition of outcome indicators; remedial measures for hypoxia. The revision of the protocol version was already informed the IRB and obtained agreement. To date, 56 participants have been recruited. This trial is still ongoing.

## Supplementary Information

**Additional file 1.** Standard Protocol Items: Recommendations for Interventional Trials (SPIRIT) 2013 Checklist.

**Additional file 2.** Trial registration Information Table.

## Data Availability

After the study is completed and the results are published, the data and trial results will be open to the public (including the participants) through email connecting with the corresponding author.

## Abbreviations

GI endoscopy: Gastrointestinal endoscopy
Group L: Lidocaine group
Group C: Control group
PSA: Procedure sedation and anesthesia
CRF: Case report forms
ASA: American Society of Anesthesiologists
