# Supplementary material for: Effects of intravenous lidocaine on hypoxemia induced by propofol-based sedation for gastrointestinal endoscopy procedures: study protocol for a prospective, randomized, controlled trial
Source: Trials. 2022 Sep 24;23:800. doi: 10.1186/s13063-022-06719-6 (PMC9509543; doi:10.1186/s13063-022-06719-6)
Supplement: Supplementary file 2 — Additional file 2. Trial registration Information Table. [file 13063_2022_6719_MOESM2_ESM.docx]

| Additional Table 2. Trial registration Information | |
| --- | --- |
| Date category | **Information** |
| Primary registry and trial identifying number | [www.chictr.org.cn](http://www.chictr.org.cn) ChiCTR2100053818 |
| Date of registration in primary registry | 30 November, 2021 |
| Secondary identifying numbers | No |
| Source(s) of monetary or material support | Beijing Administration of Traditional Chinese Medicine, PR China (Grant No. JJ-2020-62) |
| Primary sponsor | Beijing Administration of Traditional Chinese Medicine |
| Secondary sponsor(s) | No |
| Contact for public queries | Li-Xin An, MD, Professor [Email: anlixin8120@163.com] |
| Contact for scientific queries | Li-Xin An, MD, Professor, Department of Anesthesiology, Beijing Friendship Hospital, Capital Medical University, Beijing, China. |
| Public title | Intravenous lidocaine on hypoxemia induced by propofol-based sedation for gastrointestinal endoscopy procedures |
| Scientific title | Effects of intravenous lidocaine on hypoxemia induced by propofol-based sedation for gastrointestinal endoscopy procedures: study protocol for a prospective, randomized, controlled trial |
| Countries of recruitment | China |
| Health condition(s) or problem(s) studied | Hypoxemia, Oxygen-desaturation episodes |
| Intervention(s) | Active comparator: lidocaine |
|  | Placebo comparator: normal saline |
| Key inclusion and exclusion criteria | Ages eligible for study: 18-65 years; Sexes eligible for study: both; Accepts healthy volunteers: no |
|  | Inclusion criteria: ASA I–III, BMI< 30kg/m^2^, STOP-Bang < 5, heart rate [HR] > 50 beats/min without history of atrioventricular block, liver and kidney function well, without local anesthetic in the past 24 hours, without analgesics and hypnotics in the past 7 days, volunteer to participate in this research and sign the informed consent |
|  | Exclusion criteria: have participated in other clinical trials within the past four weeks, allergic to lidocaine, pregnancy or lactation, cannot understand the VAS score and cannot be scored, severe central nervous system disease and severe mental illness, taking sedative, analgesic or hypnotics in the past week, considered unsuitable to participate in this study by the investigator. |
| Study type | Interventional |
|  | Allocation: randomized; Intervention model: parallel assignment; Masking: double blind . |
|  | Primary purpose: prevention |
|  | Phase III |
| Date of first enrolment | December 2021 |
| Target sample size | 300 |
| Recruitment status | Recruiting |
| Primary outcome(s) | The incidence of oxygen-desaturation episodes |
| Key secondary outcomes | The incidence of subclinical respiratory depression, hypoxia, severe hypoxia and patients with discomfort |
